# Does Concurrent Carotid Endarterectomy with Coronary Artery Bypass Grafting Improve Outcomes in Patients with Dual Arterial Disease? A Systematic Review and Meta-Analysis

**DOI:** 10.34172/jcvtr.33605

**Published:** 2026-06-30

**Authors:** Kristine Santos, Giovanna Macanhã Scremin, Neel Patel, Amal Zakani, Mihaela Ioana Mariș, Tomasz Płonek

**Affiliations:** ^1^New Vision University School of Medicine, Tbilisi, Georgia; ^2^Medisch Spectrum Enschede, Enschede, Netherlands; ^3^Pontifical Catholic University of Paraná, Curitiba, Brazil; ^4^Mohammed V University, Faculty of Medicine and Pharmacy of Rabat, Rabat, Morocco; ^5^Center for Translational Research and Systems Medicine, “Victor Babeș” University of Medicine and Pharmacy of Timișoara, Romania

**Keywords:** Carotid endarterectomy, Coronary artery bypass grafting, Coronary artery disease, Carotid artery stenosis

## Abstract

Patients with coexisting carotid and coronary artery disease face a complex surgical decision regarding whether to undergo coronary artery bypass grafting (CABG) alone or combined with carotid endarterectomy (CEA). Evidence remains conflicting, with some studies suggesting stroke reduction from a combined approach while others report increased perioperative morbidity without a survival advantage, contributing to weak ACCF/AHA and ESC/EACTS guideline recommendations. We conducted a systematic review and meta-analysis of randomised controlled trials and propensity-matched studies comparing CABG alone versus CABG+CEA. Five studies involving 23,916 patients were included, of whom 29% underwent combined surgery. CABG+CEA was associated with a significantly higher incidence of perioperative stroke (OR 1.5; 95% CI 1.1–2.0; *P*=0.03), but no significant differences were observed in 30-day mortality, major adverse cardiovascular events, myocardial infarction, or hospital length of stay. These findings indicate that adding CEA to CABG increases stroke risk without providing a clear survival benefit, supporting guideline recommendations for a selective, risk-based strategy. Until higher-quality evidence becomes available, concurrent CEA+CABG should be reserved for carefully selected patients in whom the anticipated benefits outweigh potential harms.

## Introduction

 Coronary artery disease (CAD) and carotid artery stenosis (CAS) frequently coexist, with up to 12% of patients CABG having significant carotid lesions. ^1^ This dual pathology poses a major challenge in the optimisation of surgical revascularisation strategies, as patients with concomitant CAD and CAS are at heightened risk of perioperative stroke, a complication associated with substantial morbidity and mortality. ^2^ One strategy to mitigate this risk is the addition of CEA to CABG, performed as a single, combined procedure. However, whether this approach offers a net clinical benefit over CABG alone remains controversial.

 The American College of Cardiology Foundation and the American Heart Association (ACCF/AHA) currently offer only weak recommendations (Class IIa/b, Level C) for concurrent carotid endarterectomy (CEA) and coronary artery bypass grafting (CABG), indicating that these guidelines are based primarily on expert opinion, small studies, or inconsistent evidence rather than high-quality randomised data.^3^ Similarly, the 2018 ESC/EACTS Guidelines on Myocardial Revascularisation recommend carotid revascularisation with CEA as the first-choice strategy in patients undergoing CABG who have experienced a TIA or stroke within the past six months and have > 50% carotid artery stenosis (Class IIa, Level B).^4^ For asymptomatic patients, carotid intervention is only weakly supported (Class IIb, Level C), and is considered only in those with bilateral 70–99% carotid stenosis.^4^ The reliance on low-certainty, consensus-based recommendations across both major guidelines underscores the need for high-quality, prospective data to inform optimal management in this complex patient population.

 Numerous studies have attempted to evaluate the comparative benefit of treating coexistent carotid disease versus deferring intervention and proceeding with CABG alone; however, the findings have been inconsistent and conflicting, hence the question remains unresolved. While some researchers have advocated for the combined approach, suggesting comparable stroke risk but reduced mortality,^5^ others have reported superior outcomes with isolated CABG, including lower rates of both stroke and death.^6–8^ The central clinical question thus persists: does carotid intervention in addition to CABG improve outcomes, or does it expose patients to unnecessary perioperative risk?

 To address this question, we conducted a systematic review and meta-analysis of randomised controlled trials and propensity-matched studies comparing simultaneous CEA+CABG to isolated CABG in patients with coexisting coronary and carotid artery disease. Our objective was to clarify whether the addition of CEA improves perioperative outcomes or confers unnecessary risk, using the highest quality evidence currently available.

## Materials and Methods

 Our systematic review and meta-analysis was conducted according to the guidelines of the Cochrane Collaboration Handbook for Systematic Reviews of Interventions and the Preferred Reporting Items for Systematic Reviews and Meta-Analyses (PRISMA) statement.^9^ The review was registered in the International Prospective Register of Systematic Reviews (PROSPERO CRD420250653044).

###  Search Strategy

 The literature search was conducted using MEDLINE, Scopus, and Cochrane Library for studies written in English until from January 2000 to December 2024. The search terms included “carotid endarterectomy,” “isolated coronary artery bypass,” “synchronous CABG,” and their related words. In addition, all the references of all the included studies were also scanned for other relevant publications. The full search strategy can be found in the supplementary material.

###  Study Selection

 Titles, abstracts, and full-text articles were independently screened by two reviewers to identify eligible studies. Any discrepancies were resolved through consensus.

###  Eligibility Criteria

 Inclusion criteria were met if studies were (i) non-randomised observational studies (prospective or retrospective) or randomised controlled trials (RCT); (ii) enrolled patients with dual arterial disease; (iii) directly comparing CCC and isolated CABG; and (iv) provided quantitative data on at least one of the clinical outcomes of interest. Exclusion criteria were as follows: (i) Studies involving animal models; (ii) case reports, reviews, editorials, or studies that were lacking in data for comparative analysis or were based on studies without control groups; (iii) and single-arm studies which failed to show direct comparison of CCC and isolated CABG.

 To improve clarity and reproducibility, we defined “dual arterial disease” using quantitative criteria consistent with the thresholds applied in the included studies. Specifically, carotid artery disease was defined as ≥ 50% stenosis (symptomatic or asymptomatic) measured by duplex ultrasound, computed tomography angiography, or conventional angiography. Coronary artery disease requiring surgical revascularisation was defined as ≥ 70% stenosis in at least one major epicardial coronary artery or ≥ 50% left main stenosis. These thresholds reflect the operative definitions used across the included trials and propensity-matched studies. Where individual studies did not explicitly report stenosis severity, we relied on their stated inclusion of clinically significant carotid and coronary artery disease as defined by their institutional criteria.

###  Data Extraction and Preparation

 Data extraction was conducted by two reviewers independently. The extracted data was documented in a structured spreadsheet with any discrepancies between reviewers were resolved through discussion and consensus. When studies lacked complete summary statistics or missing data, we used conventional statistical procedures to generate the required measurements. All data conversions such as converting medians and interquartile ranges to means and standard deviations were done as originally described by Wan et al.^10^ Where it was not possible to perform conversions, studies were excluded from the analysis.

###  Outcomes

 Our meta-analysis assessed five key outcomes: mortality, hospital length of stay (LOS), incidence of permanent neurological deficits (PND), myocardial infarction (MI), and composite major adverse cardiovascular events (MACE). For the purposes of this meta-analysis, MACE were defined as a composite outcome including one or more of the following: perioperative stroke, myocardial infarction (MI), all-cause mortality, transient ischaemic attack (TIA), coma, or unplanned cardiovascular readmission, where reported. Given the variability in definitions across included studies, we accepted each study’s operational definition of MACE but aimed to harmonise the outcome by including studies that incorporated at least two of the core cardiovascular endpoints (stroke, MI, or death) within the composite measure.

 The primary outcomes were mortality and PND incidence. Mortality was defined as in-hospital or 30-day death. These were chosen as the primary outcomes because they represent the most clinically significant and irreversible adverse events following surgical revascularisation. Mortality directly reflects the ultimate failure of perioperative management and is regarded as an important endpoint in cardiac and vascular surgery. Similarly, PND captures the burden of serious, life-altering neurological injury, which is a major concern in patients undergoing combined CEA and CABG, given the known risks of cerebrovascular complications.^11^

###  Statistical Analysis

 Mean differences (MD) with their 95% confidence intervals (CI) were calculated for continuous variables using RevMan version 8.13.0. Our meta-analysis employed a Mantel-Haenszel random-effects model to account for study variance and the expected heterogeneity of the studies.^12^ To minimise the impact of sample size imbalances between the two cohorts, inverse variance weighting was applied, allowing larger studies to contribute proportionally more to the pooled estimates. Heterogeneity was assessed using the I^2^ statistic, with I^2^ > 50% considered to represent substantial heterogeneity.^13^ All statistical analyses were conducted using RevMan version 8.13.0.

###  Quality Assessment and Sensitivity Analyses

 The Risk Of Bias In Non-Randomised Studies of Interventions (ROBINS-I) tool was employed to assess the risk of bias in the included non-randomised studies ^14^ by two reviewers. Additionally, the risk of bias for the included RCT was evaluated via the revised tool to assess risk of bias in randomised trials (RoB 2).^15^ Any disagreements between the reviewers were resolved through a discussion of the reasons for the discrepancy. The certainty of evidence for each outcome was assessed using the GRADE (Grading of Recommendations Assessment, Development and Evaluation)^16^ approach by two independent reviewers with disagreements resolved through discussion and consensus. = A third reviewer was consulted in cases where consensus could not initially be reached. In addition, to assess the stability of the results, sensitivity analyses were also performed, including leave-one-out analyses on all primary outcomes and secondary outcomes with significant heterogeneity to check whether the findings were being influenced disproportionately by a single study.

## Results

###  Study Selection

 A total of 125 records were identified through systematic searches of PubMed, Scopus, and the Cochrane Library. After removing duplicate reports and excluding studies that did not meet inclusion criteria based on title and abstract, nine full-text articles were assessed for eligibility. Of these, four were excluded for reasons including lack of relevant outcomes, absence of a preoperative APT group, or other unspecified issues. Ultimately, five studies were included in the final meta-analysis. The study screening and selection process is illustrated in the PRISMA flow diagram (Figure 1).

**Figure 1:**
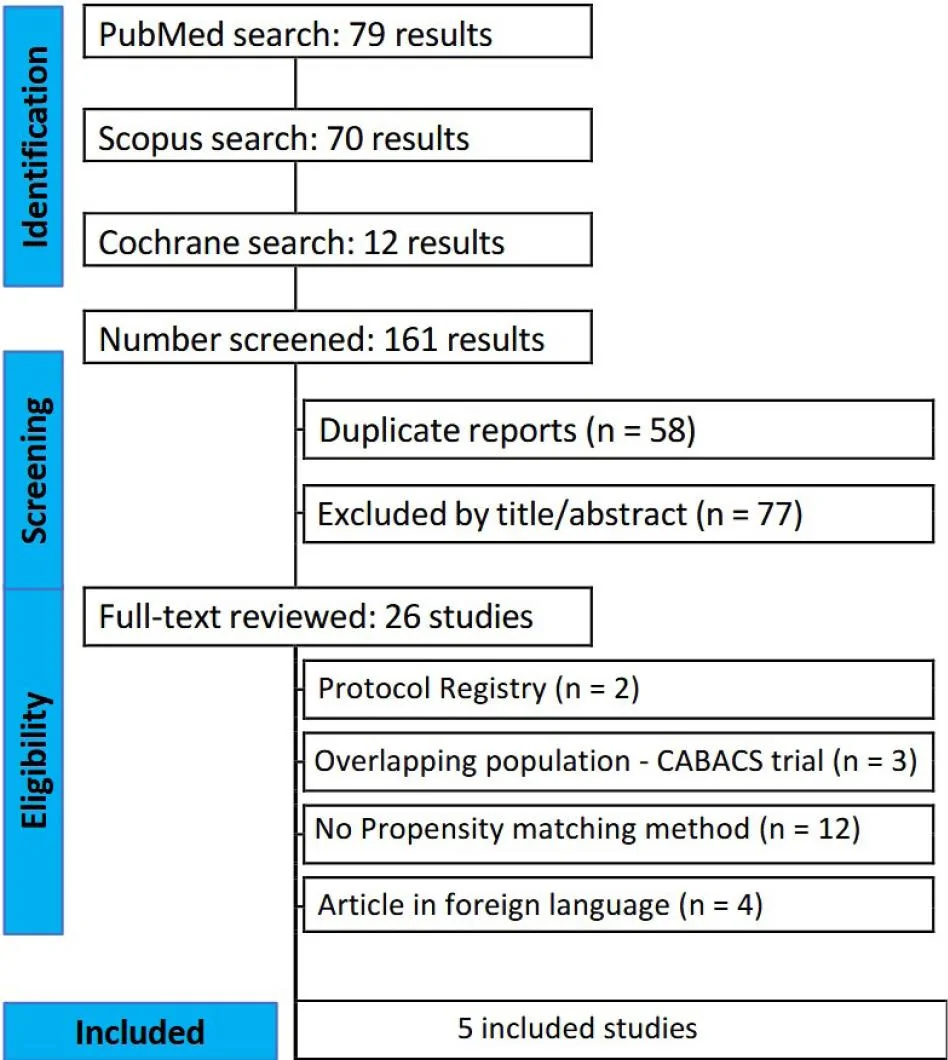
PRISMA flowchart for study screening and selection

###  Baseline characteristics

Table 1 summarises the demographic and baseline clinical characteristics of patients from the five studies encompassing a total of 23,916 patients, of whom 6,947 (29%) underwent CEA+CABG. The mean ages of the patients included in the studies ranged from 67 to 70 years. The proportion of male patients was consistently high in both treatment groups, ranging from 63% to over 83%.

**Table 1:** Baseline and demographic characteristics of patients in the included studies

**Study**	**Knipp 2022** ^8^	**Prasad 2010** ^7^	**Ricotta 2005** ^17^	**Cywinski 2006** ^6^	**Dick 2011** ^5^
Country	Germany and Czech Republic	North America	United states	United states	United states
Study design	RCT	OBS	OBS	OBS	OBS
No of patients CABG+CEA/CABG	65/62	5,732/15,757	744/744	272/272	134/134
Male sex (%) CABG+CEA/CABG	83.1/83.9	69.42/66.62	63/63	72.1 / 76.1	76/77
Age (years) mean ± SD CABG+CEA/CABG	69.7 ± 8.18/69.4 ± 8.00	69.37 ± 8.85/69.14 ± 9.57	70 ± .3/70 ± .8	69.0 ± 8.0/68.4 ± 8.9	67 ± 9.0/ 67 ± 9.0
BMI mean ± SD CABG+CEA/CABG	28.87 ± 6.72/27.76 ± 5.65	28.34 ± 5.53/28.71 ± 5.71	NR	27.6 ± 6.0/27.8 ± 6.0	NR
DM (%) CABG+CEA/CABG	NR	42.2/44.78	35/34	43/40.8	40/44
Hypertension (%) CABG+CEA/CABG	90.8/88.7	88.01/88.97	83/83	78.3/77.2	85/89
Smoker* (%) CABG+CEA/CABG	23.4/25.8	74.62/67.54	7.5/8.2	73.5/74.3	78/78
Previous TIA/CVA (%) CABG+CEA/CABG	NR	1.66/0.67	20/23	NR	22/45.3
NYHA III/IV (%) CABG+CEA/CABG	NR	17.78/20.94	15/14	NR	72/72
Previous PCI (%) CABG+CEA/CABG	NR	NR	6.70/6.70	6.3/7.4	NR
Previous Cardiac Surgery (%) CABG+CEA/CABG	NR	3.52/6.2	4.33/3.87	7.0/7.0	10/14
Severity of CAD CABG+CEA/CABG	≥ 70% stenosis	≥ 75% stenosis	< 50% to 100% stenosis	NR	NR
COPD (%) CABG+CEA/CABG	NR	4.8/4.73	23/23	10.3/8.8	22/19
Dyslipidaemia (%) CABG+CEA/CABG	72.3/69.4	78.66/80.82	NR	NR	60/58
Dialysis (%) CABG+CEA/CABG	NR	20.22/28.16	1.5/1.5	0.74/0.74	1/1

Abbreviations: BMI = body mass index, CABG = coronary artery bypass grafting; CAD = coronary artery disease; CEA = carotid endarterectomy, COPD = chronic obstructive pulmonary disease, CVA = Cerebrovascular Accident, DM = diabetes mellitus, No = number, NR = not reported, NYHA = New York Heart Association, OBS = observational studies, PCI = Percutaneous Coronary Intervention, RCT = randomised controlled trials, TIA = Transient Ischemic Attack, * = present and previous smoker

 Prevalence of common comorbidities such as hypertension (HTN) and diabetes mellitus (DM) was substantial across cohorts, with HTN exceeding 75% in all studies and DM ranging from 34% to 45% where reported. Smoking history, when recorded, varied widely (7.5% to 78%), with generally balanced distribution between groups. Prior cerebrovascular events were more frequently reported in the CEA+CABG groups, particularly in Dick et al. (22% versus 45.3%),^5^ potentially reflecting a higher-risk profile.

 Bilateral carotid artery disease was inconsistently reported and often unspecified across the included studies. Ricotta et al.^17^ explicitly stated that bilateral carotid involvement was not recorded, and no relevant data were available in the other studies included, preventing quantification of this subgroup. Regarding symptom status, only Knipp et al.^8^ included exclusively asymptomatic patients. Ricotta et al.^17^ noted that most patients were asymptomatic but did not provide a specific proportion, however, symptom status was not reported in in the other studies also limiting the ability to conduct subgroup analyses based on clinical presentation.

 Additional variables such as previous cardiac surgery, percutaneous coronary intervention (PCI), dyslipidaemia, chronic obstructive pulmonary disease (COPD), and dialysis dependence were variably reported and not consistently available across all studies. Where specified, baseline characteristics were generally well-matched between groups, though variations in reporting and missing data limit complete cross-study comparisons. These findings underscore the heterogeneity of the study populations and the need for cautious interpretation when comparing outcomes across cohorts.

###  Pooled results

 CEA+CABG was associated with a significantly higher risk of stroke compared to CABG alone [OR 1.5; 95% CI 1.3 to 1.7; *P* < 0.00001; I^2^ = 0%] (Figure 2). However, there was no significant difference in 30-day mortality between CEA + CABG and CABG alone [OR 1.3; 95% CI 0.7 to 2.2; *P* = 0.40; I^2^ = 65%] (Figure 3). In addition, the incidence of MACE [OR 1.1; 95% CI 0.7 to 1.8; p = 0.72; I^2^ = 87%] (Supplementary Figure 1), MI [OR 0.9; 95% CI 0.4 to 2.1; p = 0.87; I^2^ = 0%] (Supplementary Figure 2), and length of hospital stay [MD 1.9 days; 95% CI -0.27 to 4.0; p = 0.09; I^2^ = 93%] (Supplementary Figure 3) were comparable between the two groups. These results are summarised in Table 2.

**Figure 2:**
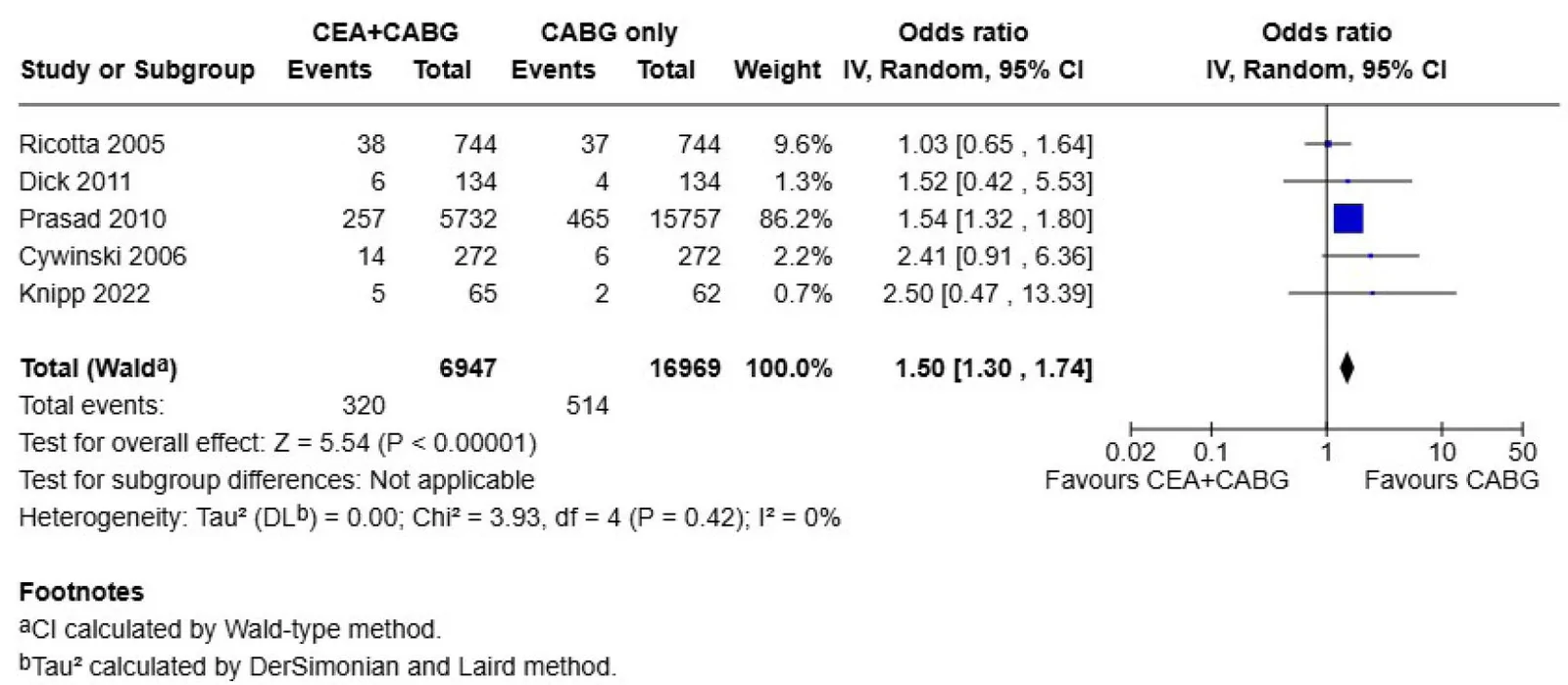
Forest plot showing significantly higher stroke risk with combined CEA and CABG compared to CABG alone [OR 1.5; 95% CI 1.3–1.7; p = 0.00001; I^2^ = 0%]

**Figure 3:**
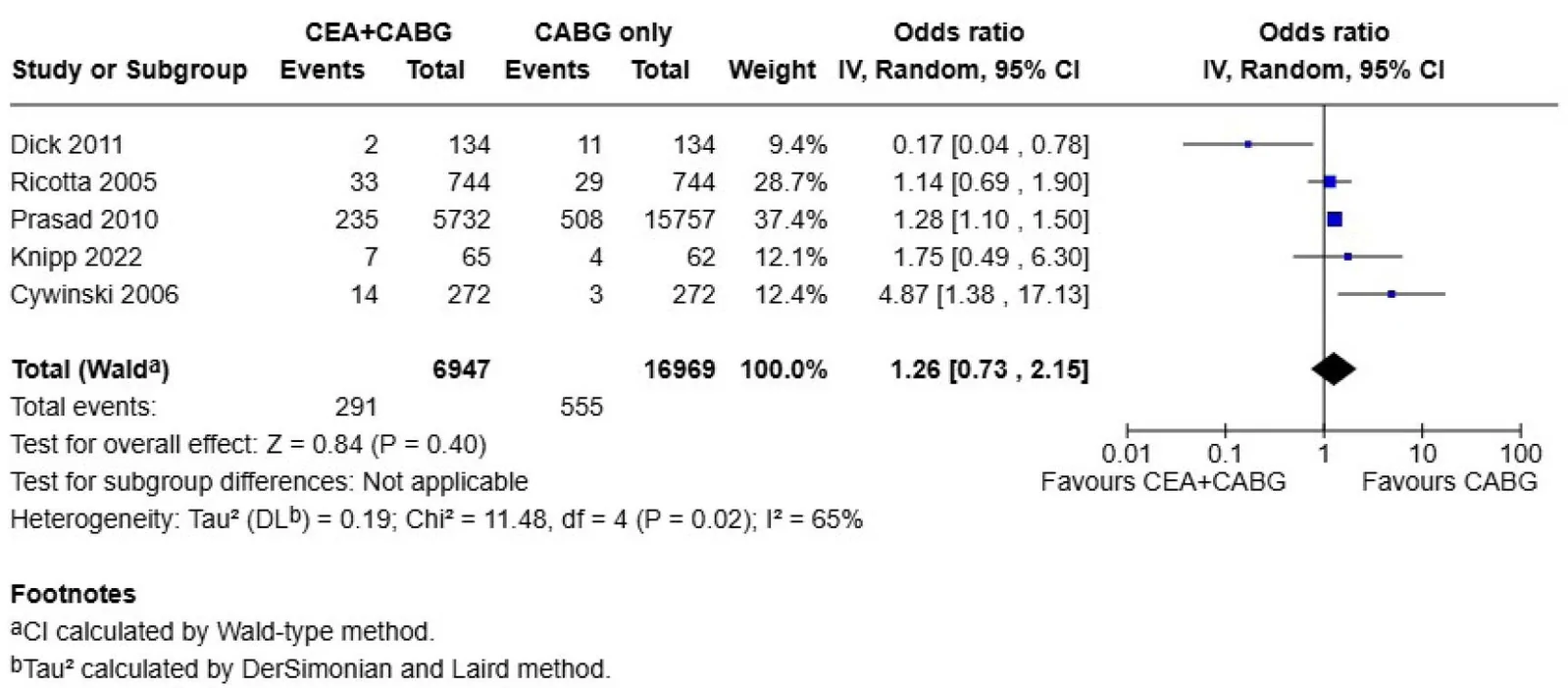
Forest plot showing no significant difference in 30-day mortality between combined CEA and CABG versus CABG alone [OR 1.3; 95% CI 0.7–2.2; p = 0.40; I^2^ = 65%]

**Table 2:** Summary of outcomes

**Outcome**	**Number of Studies**	**Number of Patients**	**Effect Estimate (95% CI, p-value)**
Stroke	5	23,916	OR 1.5; 95% CI 1.3 to 1.7; p = 0.00001; I^2^ = 0%
Composite MACE	5	23,916	OR 1.1; 95% CI 0.7 to 1.8; p = 0.8; I^2^ = 87%.
Mortality	5	23,916	OR 1.3; 95% CI 0.7 to 2.2; p = 0.4; I^2^ = 65%
Myocardial infarction	3	2,300	OR 0.9; 95% CI 0.4 to 2.1; p = 0.9; I^2^ = 0%
Length of hospital stay	3	23,521	MD 1.9 days; 95% CI -0.27 to 4.0; p = 0.1; I^2^ = 93%

CI: confidence interval; MD: mean difference; OR: odds ratio

###  Quality Assessment and Sensitivity Analyses

 The ROBINS-I tool, illustrated in Figure 4, and the ROB-2 tool, shown in Figure 5, were used to assess the risk of bias in the included studies. Among the non-randomised studies, all exhibited a low risk of bias in the classification of interventions, deviations from intended interventions, measurement of outcomes, missing data, and selection of reported results. However, moderate concerns were noted in confounding (D1) and missing data (D5) across all studies, leading to an overall moderate risk of bias.

**Figure 4:**
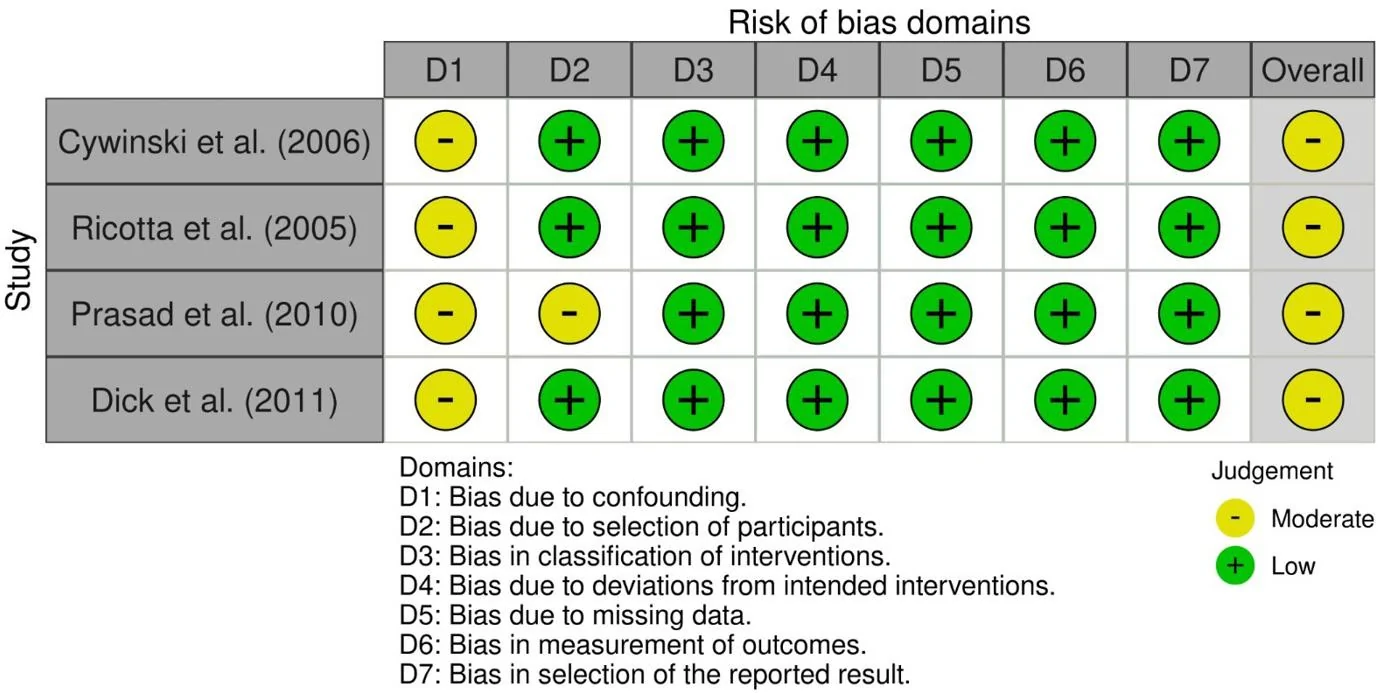
Risk of bias assessment for observational studies using ROBINS-I tool

**Figure 5:**
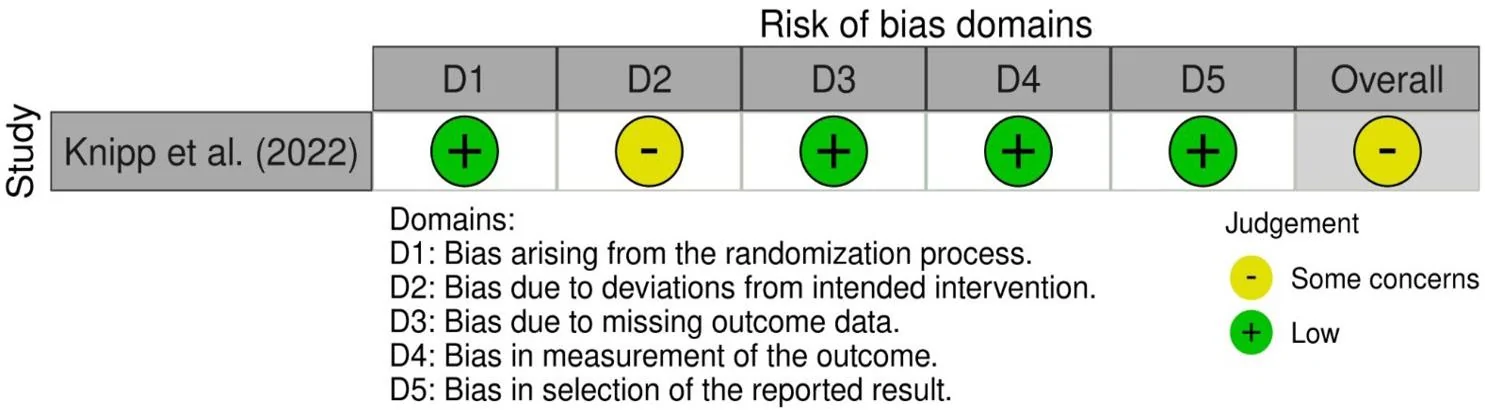
Risk of bias assessment for the randomised controlled trial using RoB-2 tool

 For the randomised study assessed using ROB-2, shown in Figure 5, a low risk of bias was observed in the randomisation process, missing outcome data, measurement of outcomes, and reporting of results. However, some concerns were identified in deviations from intended interventions (D2), contributing to an overall judgement of some concerns for this study.

 The GRADE assessment, summarised in Table 3, indicates that the certainty of evidence was low for the primary outcomes of stroke and mortality. This was primarily due to reliance on observational data and imprecision in the effect estimates. The certainty for other outcomes including incidence of myocardial infarction, MACE, and hospital length of stay, was also rated as low or very low, reflecting heterogeneity, wide confidence intervals, and the predominance of observational data.

**Table 3:** GRADE Assessment

**Outcomes**	**№ of participants (studies) Follow-up**	**Certainty of the evidence (GRADE)**	**Relative effect** **(95% CI)**	**Comments**
Overall mortality	25,207 (5 studies)	⨁⨁◯◯ Low^a^	OR 1.3 [0.7 to 2.2]; *P* = 0.4; I^2^ = 65%	Substantial inconsistency and imprecise confidence interval crossing the null
Stroke	25,207 (5 studies)	⨁⨁◯◯ Low^a^	OR 1.5 [1.3 to 1.7]; *P* = 0.00001; I^2^ = 0%	Despite inclusion of one RCT, the certainty is low as most data came from observational studies. The estimate was precise and consistent, but risk of bias remains
Myocardial Infarction	812 (3 studies)	⨁◯◯◯ Very Low	OR 0.9 [0.4 to 2.1]; *P* = 0.9; I^2^ = 0%	The certainty is very low due to serious imprecision, few events, and reliance on limited observational data, despite no observed heterogeneity
Composite MACE	25,207 (5 studies)	⨁⨁◯◯ Low^a^	OR 1.1 [0.7 to 1.8]; *P* = 0.8; I^2^ = 87%	The certainty is low due to high heterogeneity and wide imprecision
Lenght of Hospital Stay	23,324 (2 studies)	⨁◯◯◯ Very Low	MD 1.9 days [–0.27 to 4.0]; *P* = 0.1; I^2^ = 93%	High heterogeneity, confidence interval crossing the null, and relevant clinical variability.

Patient or population: patients with CAD and carotid artery stenosis Intervention: concomitant CABG and CAE (same day, not staged) Comparison: isolated CABG *The risk in the intervention group (and its 95% confidence interval) is based on the assumed risk in the comparison group and the relative effect of the intervention (and its 95% CI). CI: confidence interval; MD: mean difference; OR: odds ratio GRADE Working Group grades of evidence High certainty: we are very confident that the true effect lies close to that of the estimate of the effect. Moderate certainty: we are moderately confident in the effect estimate: the true effect is likely to be close to the estimate of the effect, but there is a possibility that it is substantially different. Low certainty: our confidence in the effect estimate is limited: the true effect may be substantially different from the estimate of the effect. Very low certainty: we have very little confidence in the effect estimate: the true effect is likely to be substantially different from the estimate of effect.

 Additional sensitivity analyses were conducted to assess the robustness of our findings. A leave-one-out approach was applied to primary outcomes and secondary outcomes exhibiting high heterogeneity, including mortality (I^2^ = 85%), incidence of PND (I^2^ = 0%), and composite MACE (I^2^ = 91%). This analysis confirmed that no single study exerted a disproportionate influence on the overall effect estimates, reinforcing the reliability of our pooled results.

## Discussion

 In our systematic review and meta-analysis of patients with concomitant coronary and carotid artery disease, we observed that performing CEA+CABG was associated with a significantly higher risk of perioperative stroke compared to CABG alone. The pooled odds suggested an approximately 1.5-fold increase in stroke risk with the combined procedure, raising important concerns about potential neurological harm. In contrast, we found no statistically significant differences in other major clinical outcomes, including all-cause mortality, MI, and MACE, between the two strategies. Hospital LOS was also similar, indicating that the addition of CEA did not appear to substantially prolong recovery. In summary, our findings suggest that CEA+CABG may increase stroke risk without offering clear benefits in terms of survival or cardiac protection and should therefore be considered with caution.

 The increased stroke risk with combined CEA+CABG may reflect compounded cerebrovascular stress from two major procedures. Carotid manipulation during endarterectomy can dislodge plaque or thrombus,^18^ while haemodynamic instability during CABG, particularly in the setting of a recently endarterectomised artery, may precipitate watershed infarcts in vulnerable cerebral territories.^19^ Prior studies have demonstrated that perioperative strokes following CABG are frequently attributable to aortic arch atheroembolism or unstable carotid plaques.^20^ Longer operative times from the combined procedure may further increase susceptibility to hypoperfusion. While these mechanisms offer a plausible rationale, they remain speculative, as stroke aetiology was not directly assessed in the included studies.

 Interestingly, despite the significantly increased risk of perioperative stroke with concurrent CEA+CABG, our analysis did not show a corresponding increase in 30-day mortality. One possible explanation is that many of these strokes may have been non-fatal, particularly if detected early and managed promptly. Additionally, selection bias may have influenced outcomes, as patients selected for the combined approach might have been better optimised preoperatively. It is also important to note that our analysis was limited to short-term outcomes; longer-term mortality differences may exist but were not captured within the 30-day timeframe assessed.

 Prior studies on the management of coexisting carotid and coronary artery disease have produced mixed results. While some observational series suggested that combined CEA+CABG may reduce stroke incidence or improve outcomes in high-risk patients, others found no benefit or even indicated potential harm. For example, in a retrospective propensity-matched analysis, Dick et al.^5^ reported similar stroke rates but significantly lower perioperative mortality in combined group (1% versus 8%), suggesting that the addition of CEA might reduce early mortality. In contrast, studies by Cywinski et al.,^6^ Prasad et al.,^7^ and Knipp et al. from the CABACS trial,^8^ associated the combined approach with worse perioperative outcomes compared to CABG alone. Cywinski et al.^6^ observed significantly higher in-hospital morbidity and mortality, while Prasad et al.^7^ found increased rates of neurological complications and all-cause mortality among patients undergoing simultaneous CEA+CABG. Similarly, the CABACS trial reported an almost twofold higher composite rate of nonfatal stroke or death within 30 days in the CEA+CABG cohort.^8^ These discrepancies likely stem from small sample sizes, heterogeneous designs, variations in symptom status, and differing surgical expertise. Nonetheless, when synthesised, the current evidence suggests that the addition of CEA increases neurological risk without clear survival or cardiac benefit.

 While our study offers important insights, it does not imply that carotid intervention has no role in CABG patients. Certain high-risk groups, including those with recent TIA or critical bilateral carotid stenosis, may still benefit from revascularisation. However, concurrent surgery is not the only strategy. Staged procedures or less invasive alternatives, such as carotid artery stenting (CAS), may be safer depending on clinical and anatomical factors. These alternative approaches, as well as the optimal timing of carotid revascularisation relative to CABG, remain critical areas for future investigation to determine the safest and most effective patient-specific management strategies for those with dual arterial disease. Moreover, clinical decision-making should be guided by a multidisciplinary team, including cardiac surgeons, vascular surgeons, neurologists, and anaesthesiologists, carefully weighing the urgency of CEA, neurologic symptom status, and overall surgical risk.

 A prior meta-analysis by Tsukagoshi et al.^21^ evaluated 25 studies (23 observational and 2 randomised), comprising 32,473 patients over a 46-year period, and compared five carotid-CABG treatment strategies: (i) combined CEA and CABG (62% of patients), (ii) staged CEA followed by CABG (21%), (iii) CABG followed by CEA (1%), (iv) combined CAS and CABG (4%), and (v) CABG alone (12%). The analysis focused on perioperative stroke, death, and myocardial infarction (MI). The primary finding was that none of the carotid intervention strategies conferred a significant advantage over isolated CABG in reducing perioperative stroke, mortality, or MI. Although staged CEA prior to CABG was associated with a lower rate of stroke or TIA, it was offset by an increased risk of perioperative mortality and MI. Conversely, the CAS+CABG approach was associated with a higher stroke/TIA rate but lower mortality. Overall, the authors concluded that none of the combined or staged carotid intervention strategies improved 30-day outcomes compared with CABG alone.

 It should be noted that while the meta-analysis by Tsukagoshi et al. offers a broad overview of five different carotid-CABG strategies, several important limitations distinguish it from our more focused and methodologically rigorous approach. Their analysis, spanning 46 years and including 25 studies, was predominantly composed of heterogeneous, non-risk-adjusted observational data, thereby limiting the ability to draw robust causal inferences and reducing its clinical applicability. In contrast, our meta-analysis directly compares simultaneous CEA+CABG with isolated CABG, restricting inclusion to randomised controlled trials and rigorously propensity-matched studies published after year 2000. This design reflects modern surgical practices and enhances internal validity by better controlling for confounding factors. By narrowing the clinical question and elevating the quality of included evidence, our analysis provides a more precise and clinically relevant understanding of the perioperative risks and potential benefits associated with combined CEA and CABG.

 The clinical implications of these findings are that routine concurrent CEA during CABG should generally be avoided, particularly in patients with asymptomatic or moderate carotid stenosis (< 70%). In these cases, the stroke risk from the carotid lesion alone is low, while the procedural risks of CEA are significant.^21^ In most cases the 1-year stroke or death risk is higher with CEA (~2%) or CAS (~4%) than with intensive medical therapy (~0.5%),^22^ supporting a conservative approach in most asymptomatic patients. Thus, prophylactic CEA in this setting may expose patients to unnecessary risk without a proven benefit. For most asymptomatic individuals, isolated CABG alongside optimal medical therapy including statins, antiplatelets, and tight blood pressure control may offer a safer and more appropriate strategy.

 Our findings align with the cautious stance adopted by both ACCF/AHA and the ESC/EACTS guidelines, which provide only weak, low-level recommendations for carotid revascularisation in CABG patients. The ACCF/AHA assigns a Class IIa or IIb recommendation based on Level C evidence,^23^ reflecting reliance on expert opinion and limited observational data rather than robust randomised controlled trials. Similarly, the ESC/EACTS guidelines recommend carotid revascularisation for patients with a history of TIA and high-grade stenosis (Class I, Level C), but advise revascularisation for asymptomatic patients only in highly selected cases (Class IIb, Level C).^24^ By showing no survival or cardiac benefit and an increased stroke risk with concurrent CEA+CABG, our analysis reinforces the rationale for an individualised, risk-based approach and underscores the need for further high-quality trials to guide clinical decision-making.

 Our study represents the most comprehensive analysis to date focused exclusively on high-quality comparative data for CEA+CABG versus CABG alone. By including only randomised trials and propensity-matched studies, we minimised confounding and improved internal validity. With nearly 24,000 patients from multiple centres, the analysis was adequately powered to detect even infrequent outcomes such as stroke. We employed a random-effects model to account for both within- and between-study heterogeneity and applied inverse variance weighting to ensure that larger studies contributed proportionally more to the pooled estimates.

 Nevertheless, several limitations must be acknowledged. Only five studies met our inclusion criteria, highlighting the scarcity of high-quality randomised data. Although the pooled sample size was large, only one study was an RCT, with the remainder being propensity-matched observational studies; thus, residual confounding remains possible, particularly for unmeasured factors such as surgeon expertise or lesion complexity. Notably, the certainty of evidence for all outcomes was rated Low or Very Low using the GRADE framework, reflecting the predominance of observational data, the limited size of the single RCT, and imprecision in several estimates. Consequently, confidence in the effect sizes is limited, and the findings should be interpreted with caution.Reporting of key anatomical and clinical variables across the included studies were also limited. Specifically, the prevalence of bilateral carotid artery disease was often unreported. Ricotta et al.^17^ explicitly stated that bilateral lesions were not documented, and Cywinski et al.^6^, Dick et al.^5^, Knipp et al.^8^, and Prasad et al.^7^ similarly did not provide this information. This precluded analysis of how bilateral disease may have influenced outcomes. Similarly, symptom status was variably defined. Knipp et al. (CABACS trial)^8^ limited inclusion to patients with asymptomatic carotid stenosis, while Ricotta et al.^17^ suggested most were asymptomatic but gave no precise data. Cywinski et al.^6^, Dick et al.^5^, and Prasad et al.^7^ did not define symptom status at all. This lack of standardisation hindered subgroup analyses and limited our ability to assess differential outcomes between symptomatic and asymptomatic patients.Other sources of heterogeneity included differences in surgical techniques, such as on-pump (ONCAB) versus off-pump CABG (OPCAB), which may impact haemodynamics and embolic risk.

 While our meta-analysis did not stratify outcomes by pump strategy, ONCAB versus OPCAB, due to limited reporting in the included studies, existing literature offers insights. Fareed et al.^25^ analysed 324 patients undergoing synchronous CEA and OPCAB, reporting an operative mortality of 1.5% and a combined stroke or death rate of 2.2%, figures that compare favourably with outcomes typically seen in on-pump procedures. However, the authors acknowledged that this cohort was likely highly selected and small, with possible publication bias, and the proportion of patients undergoing a no-touch technique was not specified. Support for this strategy comes from Emmert et al., who reported a stroke rate of 0.7% in patients undergoing OPCAB with a no-touch aortic technique, compared to 2.3% with partial aortic clamping.^26^ These findings suggest that in patients with significant carotid and coronary artery disease, especially those at high risk of aortic atheroembolism, combining CEA with OPCAB, no-touch aortic technique may offer a safe alternative.

 Furthermore, outcome definitions were not fully standardised across studies particularly for MACE which was variably defined or inconsistently reported. Some studies used it as a composite outcome but differed in the inclusion of events such as stroke, MI, death, or other complications, while others, like Knipp et al. (CABACS trial)^8^ used a composite of nonfatal stroke or death without labelling it “MACE”. In Ricotta et al.^17^, Prasad et al.^7^, Dick et al.^5^ and Cywinski et al.^6^ studies the term MACE was referenced but not uniformly described, with inconsistent inclusion of events like TIA or readmission. The heterogeneity for both the MACE (I^2^ = 87%) and hospital LOS (I^2^ = 93%) was extremely high, indicating fundamental and unexplained differences across the included studies. This likely reflects variation in perioperative management, institutional practices, and, for MACE, markedly inconsistent outcome definitions as discussed previously. As a result, the statistical combination of these outcomes is unreliable, and the pooled estimates should be interpreted with caution. Additionally, our analysis focused exclusively on perioperative and short-term outcomes. We did not capture long-term endpoints such as late stroke, readmission, or survival. It remains possible that prophylactic CEA could provide long-term stroke prevention benefits not evident in short-term data, particularly in patients with high-grade carotid lesions, but none of the included studies provided extended follow-up to explore this. Finally, potential publication bias must also be considered. High-risk procedures may be more frequently reported by centres of excellence, whereas less favourable outcomes may be underrepresented in the literature. We were unable to formally assess publication bias, as the inclusion of only five studies falls below the minimum of ten studies recommended by Cochrane guidelines for reliable funnel plot analysis.

 Our study highlights the need for further research to guide management in patients with concomitant carotid and coronary artery disease. A large, multicentre randomised trial directly comparing all major surgical strategies including concurrent CEA+CABG, staged revascularisation, and isolated CABG is needed with stratification by stenosis severity and symptom status. Previous efforts, such as the CABACS trial, were underpowered due to recruitment challenges, leaving key questions unresolved. Until more definitive data emerges, clinicians must rely on the best available, albeit imperfect, evidence from meta-analyses like ours. Beyond surgical techniques, alternative strategies also merit investigation. Carotid artery stenting (CAS), either staged or performed concomitantly with CABG, may offer a less invasive option that reduces operative time and avoids neck dissection. While early experiences with staged CAS+CABG have shown mixed results and raised concerns about peri-procedural stroke,^21^ ongoing advancements in stent design and cerebral protection may enhance its safety in high-risk patients. Similarly, aggressive medical management, optimising blood pressure, antiplatelet therapy, and statin use with vigilant neurologic monitoring, could provide a safer alternative to routine carotid intervention. However, these strategies remain to be formally evaluated in prospective controlled trials.

## Conclusion

 In summary, our systematic review and meta-analysis suggests that concurrent CEA+CABG increases perioperative stroke risk without clear short-term survival or cardiac benefit. These findings reinforce and strengthen the current ACCF/AHA and ESC/EACTS guidelines, which provide only Class IIa/b recommendations based on low-certainty (Level B/C) evidence. Our results support a selective, individualised approach, reserving combined CEA+CABG for carefully selected patients following multidisciplinary evaluation, where the potential benefits clearly outweigh the risks. However, the conclusions are tempered by important limitations, including high heterogeneity, the limited number of available studies, low certainty of evidence across all outcomes, and the inability to perform key subgroup analyses. These gaps underscore the pressing need for a large, adequately powered randomised controlled trial to definitively address this clinical question.

## Competing Interests

 The authors declare that they have no competing interests.

## Ethical Approval

 This study is a systematic review and meta-analysis of published data. No new patient data were collected, and ethical approval was therefore not required.

## Supplementary Files

Supplementary file 1 contains Figures S1-S3.
