# Supplementary material for: Does Concurrent Carotid Endarterectomy with Coronary Artery Bypass Grafting Improve Outcomes in Patients with Dual Arterial Disease? A Systematic Review and Meta-Analysis
Source: J Cardiovasc Thorac Res. 2026 Jun 30;18(2):93–102. doi: 10.34172/jcvtr.33605 (PMC13677610; doi:10.34172/jcvtr.33605)
Supplement: Supplementary file 1 [file jcvtr-18-93-s001.pdf]

J Cardiovasc Thorac Res 2026;18(2):93-S1
doi:10.34172/jcvtr.33605
https://jcvtr.tbzmed.ac.ir

Full search strategy

("carotid endarterectomy" OR CEA OR "carotid stenosis" OR "carotid artery surgery" OR "carotid surgery" OR "carotid revascularization" OR "carotid revascularisation") AND ("coronary artery bypass grafting" OR CABG OR "coronary bypass" OR "coronary artery bypass" OR "myocardial revascularization" OR "myocardial revascularisation" OR "cardiac bypass surgery") AND ("isolated coronary artery bypass" OR "isolated CABG" OR "CABG alone" OR "grafting alone" OR "CABG without CEA" OR "bypass alone" OR "standalone CABG")

Supplementary Figure 1 Composite MACE – No significant difference in major adverse cardiovascular events (MACE) between CEA+CABG and CABG alone [OR 1.1; 95% CI 0.7 to 1.8; p = 0.72; I² = 87%].

	CEA+CABG		CABG only			Odds ratio
Study or Subgroup	Events	Total	Events	Total	Weight	IV, Random, 95% CI
Dick 2011	44	134	74	134	20.4%	0.40 [0.24 , 0.65]
Ricotta 2005	63	744	69	744	22.6%	0.90 [0.63 , 1.29]
Prasad 2010	391	5732	754	15757	25.2%	1.46 [1.28 , 1.65]
Cywinski 2006	41	272	24	272	19.8%	1.83 [1.07 , 3.13]
Knipp 2022	12	65	6	62	12.0%	2.11 [0.74 , 6.04]
**Total (Wald[a])**		**6947**		**16969**	**100.0%**	**1.10 [0.67 , 1.81]**
Total events:	551		927			

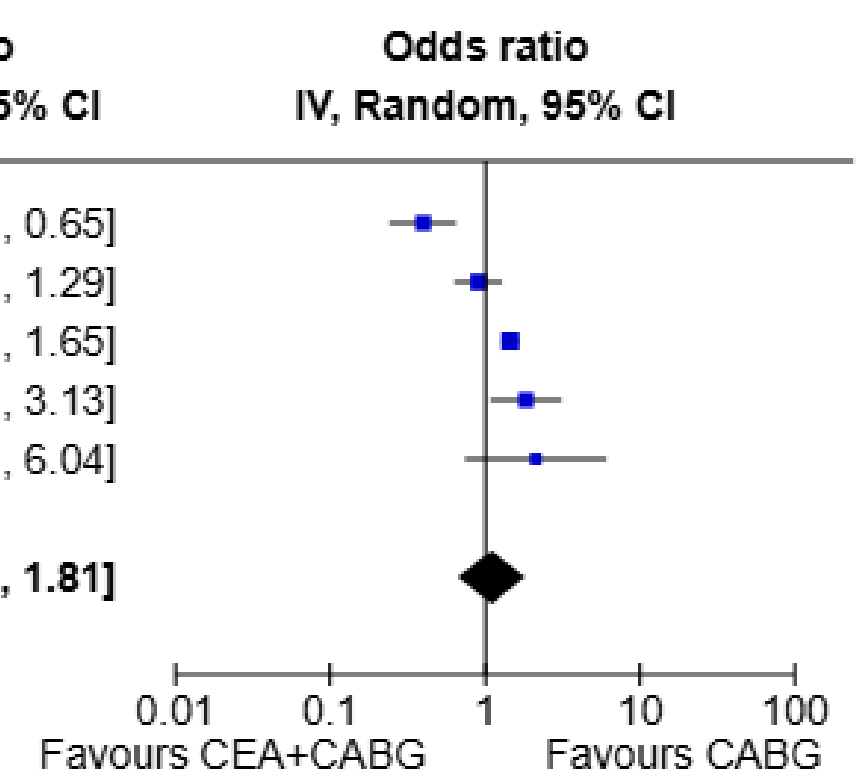

Test for overall effect: Z = 0.36 (P = 0.72)
Test for subgroup differences: Not applicable
Heterogeneity: Tau² (DL[b]) = 0.25; Chi² = 31.45, df = 4 (P < 0.00001); I² = 87%

Footnotes
[a]CI calculated by Wald-type method.
[b]Tau² calculated by DerSimonian and Laird method.

Supplementary Figure 2 Myocardial Infarction – No significant difference in perioperative myocardial infarction risk between CEA+CABG and CABG alone [OR 0.9; 95% CI 0.4 to 2.1; p = 0.87; I² = 0%].

Study or Subgroup	CEA+CABG Events	CEA+CABG Total	CABG only Events	CABG only Total	Weight	Odds ratio IV, Random, 95% CI
Ricotta 2005	4	744	8	744	43.0%	0.50 [0.15 , 1.66]
Dick 2011	1	134	1	134	8.1%	1.00 [0.06 , 16.15]
Cywinski 2006	8	272	5	272	48.9%	1.62 [0.52 , 5.01]
**Total (Wald[a])**		**1150**		**1150**	**100.0%**	**0.94 [0.43 , 2.06]**
Total events:	13		14			

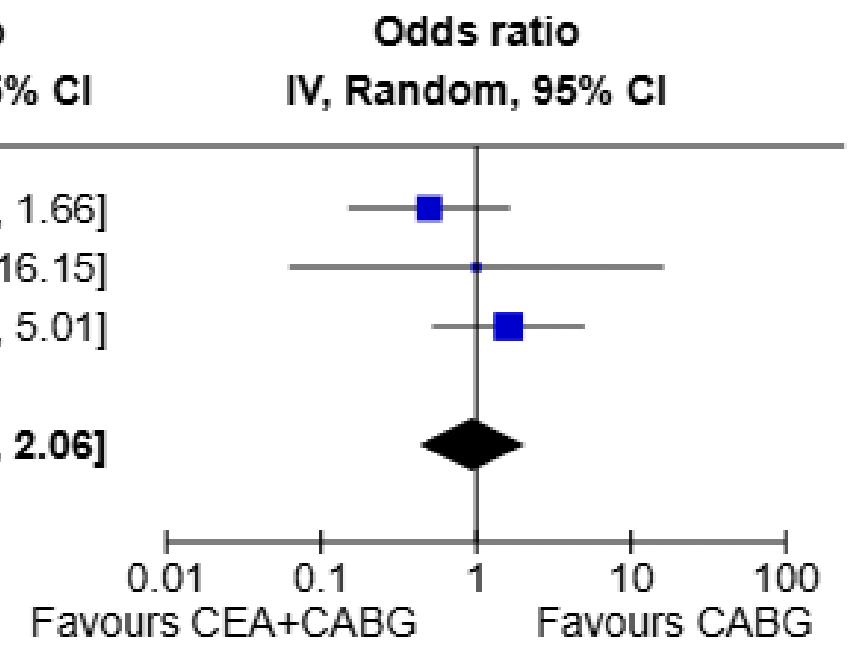

Test for overall effect: Z = 0.16 (P = 0.87)
Test for subgroup differences: Not applicable
Heterogeneity: Tau² (DL[b]) = 0.00; Chi² = 1.96, df = 2 (P = 0.37); I² = 0%

Footnotes
[a]CI calculated by Wald-type method.
[b]Tau² calculated by DerSimonian and Laird method.

Supplementary Figure 3 Length of Hospital Stay – CEA+CABG showed a non-significant trend towards longer hospital stay [MD 1.9 days; 95% CI -0.27 to 4.0; p = 0.09; I² = 93%].

Study or Subgroup	CEA+CABG Mean [days]	CEA+CABG SD [days]	CEA+CABG Total	CABG only Mean [days]	CABG only SD [days]	CABG only Total	Weight	Mean difference IV, Random, 95% CI [days]
Cywinski 2006	12	7.5	272	9	5.22	272	46.9%	3.00 [1.91 , 4.09]
Prasad 2010	11.31	8.15	5732	10.48	8.11	15757	53.1%	0.83 [0.58 , 1.08]
**Total (Wald[a])**			**6004**			**16029**	**100.0%**	**1.85 [-0.27 , 3.97]**

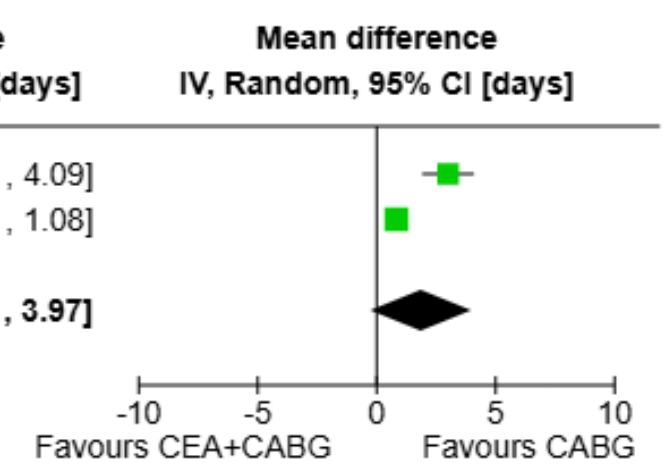

Test for overall effect: Z = 1.71 (P = 0.09)
Test for subgroup differences: Not applicable
Heterogeneity: Tau² (DL[b]) = 2.19; Chi² = 14.59, df = 1 (P = 0.0001); I² = 93%

Footnotes
[a]CI calculated by Wald-type method.
[b]Tau² calculated by DerSimonian and Laird method.
